# Talking to patients with heart failure about end of life

**DOI:** 10.1002/ejhf.1321

**Published:** 2018-10-08

**Authors:** Ross T. Campbell, Mark C. Petrie, John J.V. McMurray

**Affiliations:** ^1^ BHF Cardiovascular Research Centre University of Glasgow Glasgow Scotland, UK; ^2^ Golden Jubilee National Hospital Glasgow Scotland, UK

## Introduction

Patients with heart failure (HF), particularly those admitted to hospital, have a reduced life expectancy.^1^ When asked to consider their preferred place for end‐of‐life (EOL) care if recovery appears unlikely, many patients express a wish to spend their final days at home.^2^ Yet analysis of patients with HF in randomized controlled trials and in epidemiology studies suggests that most patients with HF die in hospital.^3^, ^4^ Whether there is a discrepancy between preferred and actual place of EOL care has not been evaluated in a prospective cohort of patients with HF. We aimed to address this question, and assess the acceptability to patients with HF (and their caregivers) of discussing EOL care.

## Methods

As part of a study assessing the palliative care needs of a near‐consecutive (i.e. consecutive patients were enrolled except during investigator vacation or other leave), prospective cohort of patients admitted to hospital with HF, patients were asked to consider their preference for place of EOL care.^5^, ^6^ Specifically, patients were asked to consider the following hypothetical scenario: ‘If you were to think about the last few days of hours of life, would you have a strong opinion or preference for where that care took place?’ Patients were given the following hypothetical options to choose from: their own home; a nursing or care home; hospital; hospice; or undecided. Patients were followed up for vital status and place of death using record linkage through Information Services Division of National Health Service, Scotland. Comparisons of categorical variables were performed using χ^2^, a *P*‐value < 0.05 was deemed statistically significant. This study was approved by the local ethics committee and all patients provided written informed consent.

## Results

**Figure 1:**
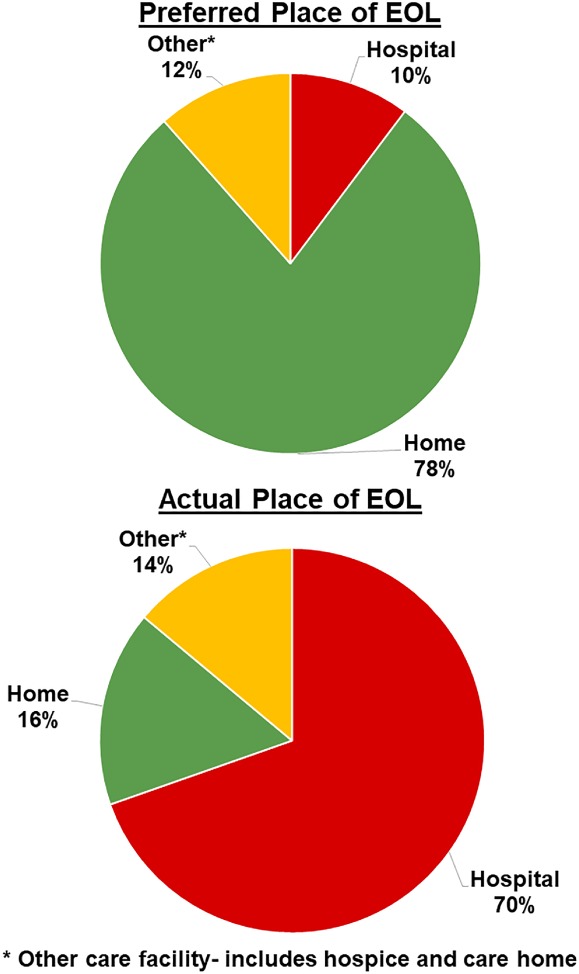
Preferred vs. actual place of death of patients who died and expressed a preference for end‐of‐life (EOL) care.

A total of 272 patients admitted to hospital with HF were enrolled (*Table* 1) between 9 January 2013 and 1 December 2014 and followed up for a median of 2.1 years. This was the first HF presentation for 152 (56%) patients and 256 of the total (94%) provided an answer to the above EOL question (*Table* 2). Most expressed a wish to spend their EOL at home. One quarter of patients did not have a strong opinion regarding EOL care location. The proportion of patients with a new diagnosis answering the EOL preference question (92%) was similar to the proportion with an existing diagnosis answering this question (97%). There was no significant difference in preference for place of EOL between those with a previous diagnosis of HF and those with a first HF presentation, although the numbers for some of the options were small (*Table* 2). The majority in the new diagnosis group (63%) and existing diagnosis group (60%) chose home or were undecided (18% and 23%, respectively). Of the 16 patients who declined to answer the EOL question, only one patient expressed an objection to being asked about EOL care preferences. Overall, 103 (38%) patients died during follow‐up, with location of death available for all patients. Of these 103 deaths, 70 (68%), 18 (18%), and 15 (15%) were in hospital, home, or another destination (including hospice or care home), respectively. Location of EOL for the 79 patients who both expressed a preference for place of EOL and died during follow‐up is shown in *Figure* 1. The distribution of place of death for these patients, who had expressed an EOL care preference, was similar to the overall group which did not survive, with 55 (70%) dying in hospital, 13 (16%) dying at home, and 11 (14%) dying at another location. Only 19 of 79 patients died in their preferred place of EOL.

**Table 1:** Baseline characteristics

*n*	272
Age, years	76 [70–82]
Female sex	128 (47)
SBP, mmHg	134 [118–155]
NYHA class	
II	82 (30)
III	141 (52)
IV	49 (18)
Previous HF diagnosis	120 (44)
Hypertension	184 (68)
Myocardial infarction	111 (41)
Atrial fibrillation	144 (53)
TIA/CVA	52 (19)
Diabetes	89 (33)
COPD	69 (25)
ICD/CRT	12 (4)
BNP, pg/mL	724 [420–1405]
eGFR, mL/min/1.73 m^2^	62 [40–82]
Hb, g/L	122 [109–136]
EF, %	38 [25–54]
EF ≥ 50%	89 (33)
Admission medications	
ACEi/ARB	148 (54)
Beta‐blocker	152 (56)
MRA	23 (8)

Values are expressed as *n* (%), or median [interquartile range].

ACEi, angiotensin‐converting enzyme inhibitor; ARB, angiotensin receptor blocker; BNP, B‐type natriuretic peptide; COPD, chronic obstructive pulmonary disease; CRT, cardiac resynchronization therapy; CVA, cerebral vascular accident; EF, ejection fraction; eGFR, estimated glomerular filtration rate; Hb, haemoglobin; HF, heart failure; ICD, implantable cardioverter defibrillator; MRA, mineralocorticoid receptor antagonist; NYHA, New York Heart Association; SBP, systolic blood pressure; TIA, transient ischaemic attack.

**Table 2:** Details of answers to preferred place of end of life scenario

	All patients (*n* = 272)	Previous HF diagnosis (*n* = 120)	First HF presentation (*n* = 152)	*P*‐value
Provided answer to EOL scenario	256 (94)	116 (97)	140 (92)	0.11
Answer to EOL scenario				
Home	157 (61)	69 (60)	88 (63)	0.05
Hospital	17 (7)	12 (10)	5 (4)	
Care facility	24 (9)	6 (5)	18 (13)	
Hospice	6 (2)	2 (2)	4 (3)	
Undecided	52 (20)	27 (23)	25 (18)	

Values are expressed as *n* (%).

EOL, end of life; HF, heart failure.

## Discussion

This is the first study of a prospective and near‐consecutive cohort of patients with HF to compare preferred with actual place of death. Our first important finding is that patients with HF, even with a first presentation, are willing to discuss EOL care. There was no difference in proportion of patients willing to discuss EOL between those with and without a prior diagnosis of HF, or the preferred place of EOL care, suggesting these conversations are applicable even during a first hospitalization for HF. Only one patient expressed concern at being asked about EOL care. The second key finding is that we found a major discrepancy between preferred and actual place of EOL care. We do not know reasons for this and clearly it is an area for further investigation. This discrepancy could reflect a lack of palliative care input or resources, limited EOL communication, or simply a disconnect between patient expectations and the reality of dying from HF. Further research may help determine how this aspect of EOL care can be improved to meet patient preferences.

### Funding

This study was supported by a British Heart Foundation Project Grant, grant number PG/13/17/30050.

**Conflict of interest**: none declared.
